# Strong Mode Coupling and Maximum Directional Absorption in Reciprocal Bianisotropic Metasurfaces

**DOI:** 10.1002/nap2.70145

**Published:** 2026-06-16

**Authors:** L. M. Máñez‐Espina, B. Amrahi, V. S. Asadchy, A. Díaz‐Rubio

**Affiliations:** ^1^ Nanophotonics Technology Center Universitat Politècnica de València València Spain; ^2^ Department of Electronics and Nanoengineering Aalto University Espoo Finland

## Abstract

Electromagnetic mode coupling plays a key role in many resonant effects in nanophotonics. This coupling is also responsible for the appearance of bianisotropy, where electric and magnetic responses become interconnected through the interaction of their respective modes. In this work, we develop a simple and general temporal coupled‐mode theory model to describe off‐diagonal chiral bianisotropy. Using quasi‐bound states in the continuum (q‐BICs), we demonstrate how to control the hybridization of modes with opposite symmetries, resulting in Rabi‐like splitting between the hybrid states in the regime of strong electromagnetic mode coupling. Beyond revealing the physical origin of the hybrid modes, our model predicts and explains the emergence of dual‐band asymmetric reflection and absorption and how to achieve the maximum directional absorption difference. The theoretical predictions are verified by full‐wave simulations, showing very good agreement with theory. Furthermore, very strong reciprocal bianisotropy is demonstrated with the use of q‐BICs in a deeply subwavelength metasurface in the optical frequency range. Our results provide a clear physical picture of the interaction process between modes, offering a compact theoretical framework for understanding and designing bianisotropic dielectric metasurfaces not only in the traditional regime but also in the strong coupling regime.

## Graphical abstract

Symmetry breaking in q‐BIC dielectric metasurfaces enables strong electromagnetic coupling between electric and magnetic modes, leading to their hybridization and Rabi‐like splitting. This mechanism produces pronounced reciprocal bianisotropy and dual‐band asymmetric absorption under opposite illumination directions, offering a compact framework for designing highly efficient, direction‐dependent light–matter interactions in nanophotonic systems.

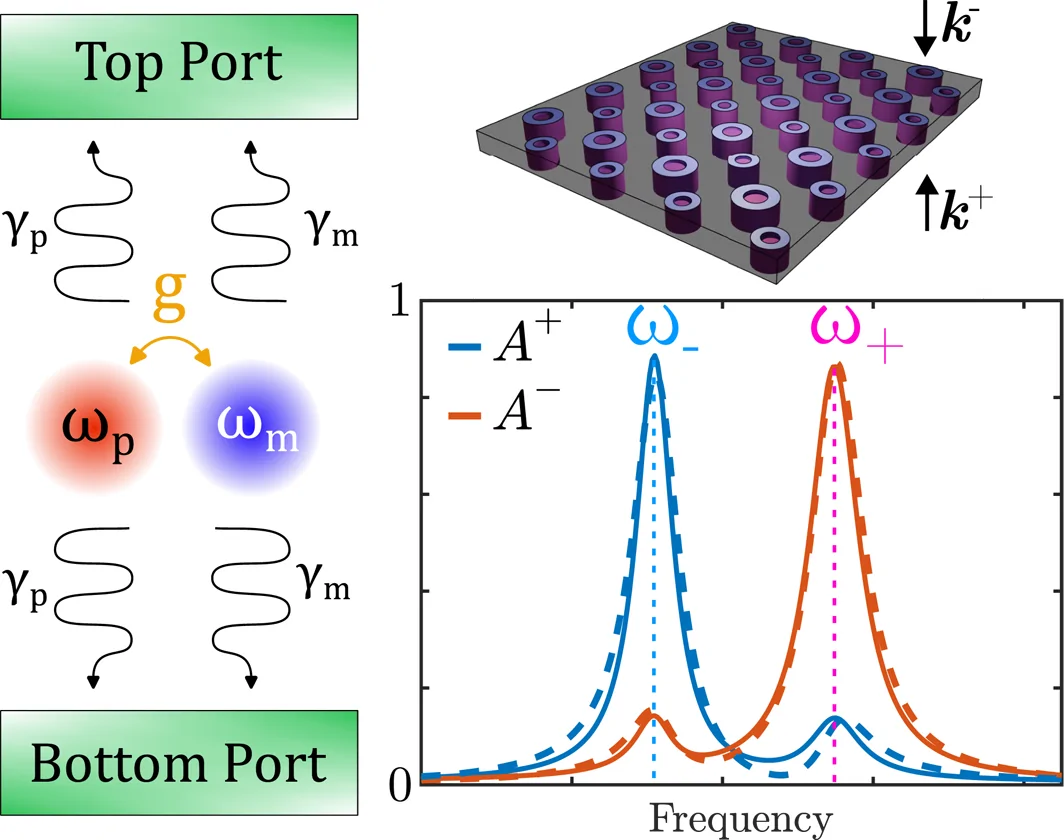

## 1 Introduction

Resonant modes in nanophotonic structures are a central topic in modern optics because they enable light–matter interactions that can give rise to a wide range of exotic effects [1, 2, 3]. When the electromagnetic field remains confined for long times inside a resonator, even weak material responses or small geometrical asymmetries can produce large observable phenomena. This confinement is commonly quantified by the quality factor (Q‐factor) of the resonance, which measures how long the energy can be stored before it is radiated or dissipated. Therefore, achieving high‐Q resonances is essential for enhancing the interaction between light and structured matter [4, 5, 6].

A major breakthrough in the quest to obtain and control high‐Q resonances in photonics was the introduction of bound states in the continuum (BICs) [7]. BICs are nonleaky resonant modes that exist within the continuum of radiative states. These modes cannot be directly excited by external plane waves and, ideally, do not radiate energy into the surrounding medium. Their practical counterparts, known as quasi‐bound states in the continuum (q‐BICs), arise when a small perturbation allows these otherwise confined modes to couple to external excitation. These modes possess finite lifetimes, and their most remarkable feature is that their Q‐factor can be tuned by controlling the strength of the perturbation [8], providing a flexible mechanism for tailoring light confinement and radiative coupling [9].

BICs can be classified according to their formation mechanism [10, 11, 12]. Among all types, the most relevant and widely studied in nanophotonics are the symmetry‐protected BICs, particularly in dielectric metasurfaces [13]. In these cases, the symmetry of certain modes is incompatible with that of the incident field, preventing any radiative coupling. Breaking the protecting symmetry enables coupling with plane‐wave excitation, transforming a BIC into a q‐BIC. The resulting Q‐factor scales inversely with the square of the perturbation strength [8, 14], $Q=B/\Delta^{2}$, where B is a constant and Δ is the characteristic size of the perturbation. Typically, in‐plane symmetry breaking is employed to achieve this controlled coupling between incident light and q‐BIC modes.

However, an even richer physical picture emerges when different resonant modes interact with each other [15]. The coupling between modes of distinct symmetry, polarization, or physical origin can profoundly modify the optical response of the system [16, 17, 18]. Such mode–mode interactions can lead to hybridized states, energy splitting, and interference phenomena that cannot be understood from single‐mode analysis [19]. In the context of metasurfaces, most studies of coupling investigate the interaction between an excitonic mode of the material and a resonant electromagnetic mode supported by the metasurface [20, 21, 22, 23, 24]. Recently, diagonal chirality, a reciprocal spatial effect that can be modeled as the electromagnetic coupling of the magnetic and electric dipoles that share directions in space, has been studied in conjunction with q‐BICs [25, 26], showing that maximum chirality can be achieved by coupling resonant modes.

Another interesting phenomenon arising from mode interaction is the electromagnetic coupling between *orthogonal* electric and magnetic dipole moments. This effect is commonly referred to as omega coupling (off‐diagonal chirality) due to the Ω‐shape of the metallic resonator, which intuitively explains this phenomenon [27, 28, 29, 30]. In optics, the same effect is usually realized by breaking out‐of‐plane symmetry with the use of a substrate or a two‐material resonator system [31, 32, 33]. To the best of our knowledge, this effect has not been studied in conjunction with BICs.

In this work, we propose a theoretical framework based on temporal coupled‐mode theory (TCMT) to describe and design optical metasurfaces that support maximum directional absorption via strong omega bianisotropy. This TCMT gives us insight into the possibilities of a dielectric structure that supports two resonances with opposite symmetries and that has broken out‐of‐plane symmetry, which allows electromagnetic coupling. Interestingly, the TCMT can demonstrate the Rabi splitting that is followed by strongly coupled resonances. Furthermore, if losses are added to the system, new venues for optical responses open. We report dual‐band asymmetric absorption, similar to what can be implemented with metallic structures [34, 35], but with opposite responses under counterpropagating excitations, directional absorption difference, and out‐of‐band transparency. Moreover, the conjunction of q‐BICs and broken out‐of‐plane symmetry allows very strong and tunable off‐diagonal bianisotropy in the optical regime.

## 2 Omega Bianisotropy and Mode Hybridization

Under plane‐wave illumination, the scattering cross‐section of a dielectric nanoparticle can be computed using the multipole expansion of scattered fields [36]. For a single cylindrical nanoparticle placed in air with diameter D, height h, and refractive index $n_{d}=3.5$ to resemble the refractive index of silicon at optical frequencies, the dipolar contributions to the scattering cross‐section are shown in Figure 1a. The particle is illuminated by a plane wave propagating along the z‐axis ($k=k_{z}u_{z}$, where $u_{z}$ is the unit vector pointing in the positive z‐axis direction), with the electric field polarized along the x‐direction. Under these conditions, a magnetic dipole moment is created in the y‐direction (represented as $m_{y}$) and an electric dipole moment along the x‐direction (represented as $p_{x}$). The scattering cross‐section is the same for $k_{z}>0$ and $k_{z}<0$, as the cylinder has mirror symmetry, $\sigma_{h}$, with respect to the xy‐plane. Furthermore, the uniaxial symmetry of the cylinder ensures the same scattering cross‐section for all polarizations. In this case, bianisotropy is forbidden by symmetry.

**FIGURE 1:**
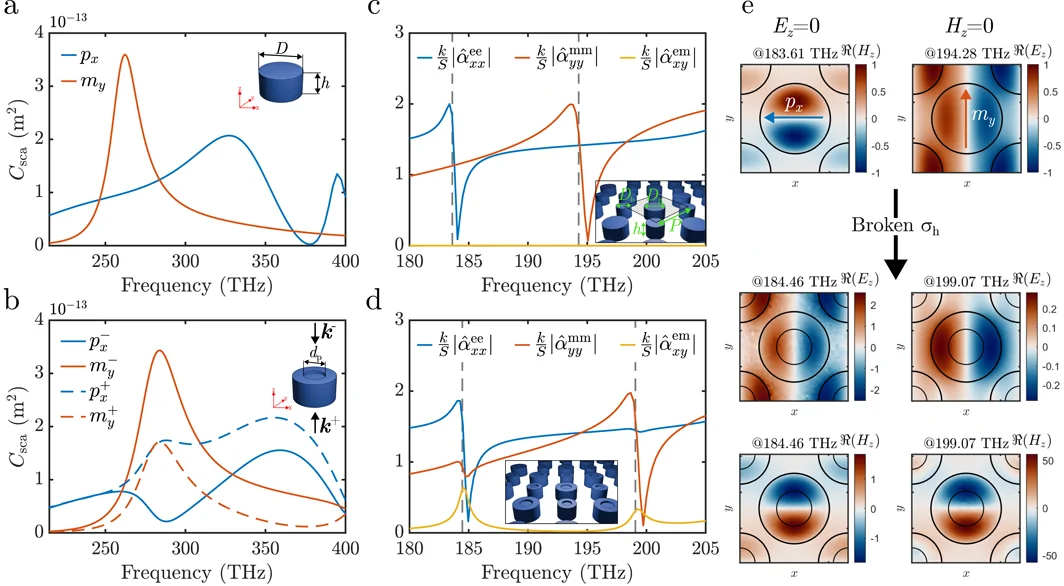
Scattering properties of off‐diagonal chiral bianisotropic particles and metasurfaces. (a) Scattering cross‐section dipole contributions of a single cylindrical nanoparticle in air with dimensions D=300 nm, h=250 nm, and refractive index $n_{d}=3.5$. (b) Scattering cross‐section of a mirror‐symmetry‐broken particle. Dipole contributions to the scattering cross‐section for different propagation directions. Same geometrical parameters as in (a) are used with a perforated hole having dimensions $h_{p}=h/2$ and $d_{p}=D/2$. (c) Absolute value of the normalized collective polarizabilities calculated for the mirror‐symmetric metasurface. In the frequency range shown, the structure holds two q‐BIC eigenmodes: a magnetic dipole and an electric dipole resonance. Geometrical parameters: P=1000 nm, D=600 nm, h=250 nm, Δ=100 nm, and $n_{d}=3.5$. Nanodisks have diameters defined as $D_{a}=D+\Delta /2$ and $D_{b}=D-\Delta /2$. (d) A metasurface with broken out‐of‐plane symmetry, holding the two hybridized resonant q‐BIC modes. Omega‐type (off‐diagonal chiral) polarizability component, $\left|{\hat{\alpha }}_{xy}^{em}\right|$, is different from zero. The perturbation, characterized by perforations on the top part of the nanodisks, has dimensions $h_{p}=20$ nm and normalized diameter $\sigma_{p}=d_{p}/D_{i}=0.5$. All other geometrical parameters are the same as in (c). (e) Top: field profiles of the out‐of‐plane components of the two mirror‐symmetric eigenmodes of the metasurface introduced in (c). Bottom: field profiles for the mirror‐symmetry‐broken metasurface shown in (d). The depicted fields are normalized with respect to the mirror‐symmetric modes $H_{z}$ and $E_{z}$. The eigenfrequencies of the modes are also depicted by vertical lines in (c) and (d).

However, in the case where $\sigma_{h}$ is broken by perforating the top part of the cylinder with depth $h_{p}$ and diameter $d_{p}$, as shown in the inset of Figure 1b, bianisotropy is enabled. This bianisotropic behavior is caused by the breaking of the out‐of‐plane mirror symmetry and can be understood in terms of group theory and the representations followed by the electromagnetic resonant modes [17, 37]. Further discussion is given in Supporting Information S1: Section 1. The scattering cross‐sections for counterpropagating plane waves are shown in Figure 1b. It can clearly be seen how, at previously resonant frequencies for the electric and magnetic dipole moments, both contributions interact. To further illustrate the discussion, we present the model for the electric and magnetic dipole moments in terms of the incident fields and the individual polarizabilities of the nanoparticle as follows [38]:

(1) $$\left(\begin{array}{c} p_{x}/\sqrt{\varepsilon_{0}}\\ m_{y}/\sqrt{\mu_{0}} \end{array}\right)=\left(\begin{array}{cc} \alpha_{xx}^{ee} & \pm \alpha_{xy}^{em}\\ \alpha_{yx}^{me} & \pm \alpha_{yy}^{mm} \end{array}\right)\left(\begin{array}{c} \sqrt{\varepsilon_{0}}\;E_{x}^{i}\\ \sqrt{\mu_{0}}\;H_{y}^{i} \end{array}\right)$$

where $\alpha^{ee}$ is the electric polarizability, $\alpha^{mm}$ is the magnetic polarizability, and $\alpha^{em/me}$ are the electromagnetic and magnetoelectric polarizabilities, all calculated for an individual particle; $E^{i}$ and $H^{i}$ are the electric and magnetic incident fields; $\varepsilon_{0}$ is the electric permittivity of free space; $\mu_{0}$ is the magnetic permeability of free space; and $p_{x}^{\pm }$ and $m_{y}^{\pm }$ are the electric and magnetic dipole moments with the superscripts ‘±’ denoting the propagation direction of the incident wave. The subscripts used in Equation (1) denote the projections of the corresponding quantities. As already mentioned, broken $\sigma_{h}$ enables bianisotropy. Specifically, the cross components of the $\alpha^{em/me}$ polarizabilities can be different from zero. Notice that, as no external bias, nonlinearity, or time modulation occurs, the structure is symmetric with respect to time reversal, and reciprocity is preserved. Reciprocity ensures an important symmetry constraint, $\alpha^{em}=-{\left(\alpha^{me}\right)}^{T}$; therefore, $\alpha_{yx}^{me}=-\alpha_{xy}^{em}$ [38, 39].

The expressions in Equation (1) describe the individual polarizabilities of a single particle in isolation. However, in a periodic array, the local field acting on each particle differs from the incident field due to the mutual interactions among all other scatterers. These interactions give rise to collective polarizabilities, which account for the multiple‐scattering and lattice effects within the metasurface. The collective polarizability tensor, $\hat{\alpha }$, can be understood as an effective response function that relates the averaged induced dipole moments to the macroscopic incident fields on the array, incorporating both the intrinsic response of the individual nanoparticle and the electromagnetic coupling mediated by the lattice [29]. The scattering parameter expressions in terms of the collective polarizabilities are given as follows [40]:

(2) $$t_{xx}^{\pm }=1-\frac{jk}{2S}\left({\hat{\alpha }}_{xx}^{\text{ee}}+{\hat{\alpha }}_{yy}^{\text{mm}}\right),$$

(3) $$r_{xx}^{\pm }=-\frac{jk}{2S}\left({\hat{\alpha }}_{xx}^{\text{ee}}-{\hat{\alpha }}_{yy}^{\text{mm}}\;\pm \;2{\hat{\alpha }}_{xy}^{\text{em}}\right),$$

with S being the area of a single unit cell, k=ω/c being the free space wavenumber, ω=2πf being the angular frequency, $c=1/\sqrt{\varepsilon_{0}\mu_{0}}$ being the speed of light in free space, j being the imaginary unit, $r_{xx}^{\pm }$ being the reflection coefficients for x−polarized to x−polarized light for propagation directions + for positive $k_{z}$ and − for negative $k_{z}$, and $t_{xx}^{\pm }$ being the transmission coefficients for the same copolarized components for positive and negative propagation directions.

Figure 1c shows the normalized collective polarizabilities for a checkerboard‐like square array of cylinders with different diameters between nearest neighbors [41, 42, 43, 44]. Diameters are defined as $D_{a}=D+\Delta /2$ and $D_{b}=D-\Delta /2$, where Δ denotes the geometrical perturbation parameter. The array is characterized by a height h and period P. Indeed, the difference in diameter between nearest neighbors is the perturbation that transforms the BIC into a q‐BIC. This specific perturbation changes the lattice from monoatomic to diatomic, bringing the dark mode at the X point of the unperturbed lattice to the Γ point of the perturbed lattice. This manipulation is usually called first Brillouin zone folding [14]. The structure is illuminated at normal incidence by an x‐polarized plane wave. It can clearly be seen that, at approximately 194 THz, the magnetic polarizability undergoes a rapid change due to a magnetic dipole‐like resonance and that the same takes place at approximately 184 THz for the electric polarizability due to an electric dipole‐like resonance. The field profiles and resonant frequencies of such resonant modes are shown in Figure 1e. Because of $\sigma_{h}$ symmetry, the eigenmodes must be either symmetric or antisymmetric with respect to the z‐axis. This leads to a clear classification between modes that have either $H_{z}=0$ or $E_{z}=0$ in the xy‐plane of symmetry. The first case corresponds to a circulation of an electric field that induces magnetic dipole moments in the cylinders with opposite directions between nearest neighbors. The second case corresponds to a magnetic field circulation inducing an electric dipole‐like moment that, as in the previous case, has opposite directions between nearest neighbors. The two modes can be classified as quasi‐orthogonal modes that do not interact, as the overlap integrals of their near‐field distributions are almost vanishing.

By applying the same out‐of‐plane perturbation as in the case for an isolated particle shown in Figure 1b to each cylinder in the array, omega‐like behavior can be induced. The drilled hole has depth $h_{p}$ and normalized diameter $\sigma_{p}=d_{p}/D_{i}$, where $D_{i}$ is the diameter of a single cylinder and takes the value $D_{a}$ for the large cylinders and $D_{b}$ for the small ones. The normalized collective polarizabilities of the metasurface holding q‐BICs with this perturbation are shown in Figure 1d. It can be clearly seen that the small perforation has introduced $\left|{\hat{\alpha }}^{em}\right|$ and that the electric and magnetic collective polarizabilities have also been perturbed. At the resonant frequency of the collective magnetic polarizability, the collective electric polarizability is affected, and conversely, at the resonant frequency of the collective electric polarizability, the collective magnetic polarizability is affected. This mixing of behaviors and electromagnetic coupling between dipole moments is also visible in the field distributions of the eigenmodes of the out‐of‐plane asymmetric metasurface, shown in Figure 1e. Both modes present a magnetic dipole and an electric dipole‐like field distribution. This is often referred to as mode hybridization [17]. The broken out‐of‐plane symmetry makes it impossible to classify the modes by their parity as symmetric and antisymmetric; both behaviors are present. The overlap integrals between the hybridized modes are nonzero, making them interact and exchange energy when they have resonant eigenfrequencies close to each other.

The use of q‐BIC resonances instead of usual resonances in our study is motivated by the versatility that the former bring in terms of radiative coupling engineering, given by their scaling law $Q=B/\Delta^{2}$ [8, 14]. Furthermore, as temporal coupled‐mode theory (TCMT) is only valid in the high‐quality‐factor regime, where the decay rates of the resonances, γ, are much smaller than their angular eigenfrequencies, ω [45, 46, 47], controlling the quality factors is a crucial matter. Therefore, the use of q‐BIC resonances offers the possibility of controlling the enhancement of the effects to be studied in the next sections and is the natural platform for proving the validity of the theoretical models developed. Further discussion can be found in Supporting Information S1: Section 2.

## 3 Strong Mode Coupling via Quasi‐Bound States in the Continuum

At microwave or radio frequencies, metals do behave as perfect conductors, which makes it possible to achieve very high confinement and avoid strong nonlocal couplings. This facilitates the modeling of the individual polarizabilities of metal elements using analytical expressions, which can then be used to calculate the collective polarizabilities of periodic structures and solve the scattering problem in terms of geometrical parameters. However, at optical wavelengths, mode confinement is much weaker. Even with the use of metals, losses and finite permittivities bound the confinement of the electric field in typical metasurfaces. Furthermore, the use of dielectric materials as substrates, such as silicon oxide, further reduces the possible field confinement granted by a lower index contrast between the structure and the host material or environment. Moreover, dielectric meta‐atoms are typically larger, and the ratio between the periodicity of the structure and the wavelength increases, enhancing nonlocal phenomena such as spatial dispersion. Because of these aspects, although modeling metasurfaces through collective polarizabilities or susceptibilities provides valuable qualitative insight, it becomes insufficient in the optical domain to provide clear insight into the actual structures required to produce the desired scattering response. Furthermore, both dipolar polarizabilities and susceptibilities become dependent on the angle of incidence unless higher‐order multipole moments are accurately considered [48, 49].

To overcome these issues, alternative analytical methods are typically employed to elucidate the physical mechanism at play. For this reason, temporal coupled‐mode theory (TCMT) has become a powerful framework to analyze the response of resonant nanostructures [45, 50], as it relates the scattering properties of the system to the properties of its eigenmodes. Over the last decades, this formalism has been extensively developed in photonics [46, 50, 51, 52, 53] and successfully applied to describe a wide variety of photonic systems, including metasurfaces supporting quasi‐bound states in the continuum. In this section, we introduce a simple yet useful approach to model omega bianisotropy in metasurfaces using TCMT. In our theory, we assume an effectively local response by taking into account only the first and second orders of spatial dispersion (including electric and magnetic dipole moments). For the considered frequency range, the influence of the quadrupole and higher‐order moments is negligible, as confirmed by the good agreement between our theory and full‐wave simulations.

Inspired by the analysis made in Section 2, we define the unperturbed system supporting two resonant modes: one that decays symmetrically—as an in‐plane electric dipole—and one that decays antisymmetrically—as an in‐plane magnetic dipole. A schematic of the model is shown in Figure 2a. The resonant structure is assumed to have uniaxial symmetry; that is, no polarization conversion is allowed, and thus, it can be modeled with only two ports in the case of normal‐incidence illumination with no diffraction orders higher than the zeroth being open. Therefore, input waves with vector $|s_{+}\rangle ={\left(s_{t+},s_{b+}\right)}^{T}$ and output waves with vector $|s_{-}\rangle ={\left(s_{t-},s_{b-}\right)}^{T}$ will only have two elements: a top (t) port and a bottom (b) port. The unperturbed resonant modes are defined by two eigenfrequencies, labeled as $\omega_{p}$ and $\omega_{m}$ for the electric and magnetic resonant modes, respectively. The decay rates that describe the total modal energy leakage are defined by $\gamma_{p}$ and $\gamma_{m}$, for the electric and magnetic resonant modes. Imposing reciprocity on the system [45, 54], the governing equations can be written, denoting $j=\sqrt{-1}$, as follows:

(4) $$\frac{da}{dt}=\left(j\Omega -\Gamma \right)\cdot a+D^{T}\cdot |s_{+}\rangle ,\;|s_{-}\rangle =C\cdot |s_{+}\rangle +D\cdot a,$$

where the amplitudes of the resonances are contained in the vector $a=\left(a_{p},a_{m}\right)$; p and m labels refer to electric and magnetic, respectively; and the resonant properties of the structure are defined in the Ω and Γ matrices as follows:

(5) $$\Omega =\left(\begin{array}{cc} \omega_{p} & g\\ g & \omega_{m} \end{array}\right),\;\Gamma =\left(\begin{array}{cc} \gamma_{p} & 0\\ 0 & \gamma_{m} \end{array}\right).\;$$

Notice that both resonances are coupled by a real coupling parameter, g, which accounts for the strength of coupling between modes, that is, the energy exchange ratio between modes [44], that depends on the out‐of‐diagonal symmetry breaking.

**FIGURE 2:**
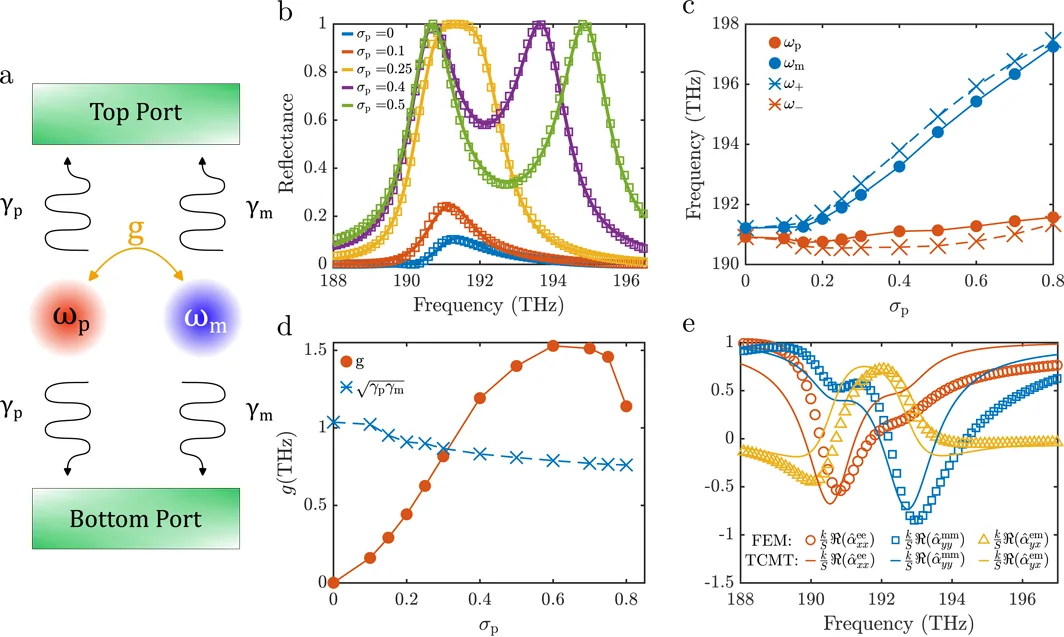
Modeling of off‐diagonal (omega) chiral bianisotropic metasurfaces with TCMT. (a) Schematic representation of the TCMT model. (b) Comparison between full‐wave and TCMT calculations of the reflectance of the metasurface for different values of the normalized diameter $\sigma_{p}=d_{p}/D_{i}$ when P=1200 nm, D=530 nm, h=263 nm, Δ=95 nm, $h_{p}=20$ nm, and n=3.5. For $\sigma_{p}=0$, the system is close to Huygens' condition with $r_{d}$ close to zero at the resonant frequency. (c) Resonant frequencies of the two hybridized modes for different values of the normalized diameter $\sigma_{p}=d_{p}/D_{i}$. The values for the fitted parameters of the unperturbed structure are also depicted, $\omega_{p}$ and $\omega_{m}$. (d) Values for the coupling strength, g, and the critical condition $g_{c}=\sqrt{\gamma_{p}\gamma_{m}}$ in terms of the normalized diameter. (e) Real parts of the collective electric, magnetic, and magnetoelectric polarizabilities as functions of the TCMT parameters, represented by solid lines, compared with the corresponding values obtained from FEM simulations, represented by points.

The D matrix represents the coupling between modes and ports. The C matrix models the direct coupling (direct process or background process) between input and output waves. They are defined as follows:

(6) $$D=\left(\begin{array}{cc} d_{\text{tp}} & d_{tm}\\ d_{bp} & d_{bm} \end{array}\right),\;C=e^{j\phi }\left(\begin{array}{cc} r_{d} & jt_{d}\\ jt_{d} & r_{d} \end{array}\right).$$

The elements of the coupling matrix D, due to the symmetrical and antisymmetrical nature of the unperturbed resonances, follow $d_{tp}=d_{bp}$ and $d_{tm}=-d_{bm}$, where t and b refer to the top and bottom ports and p and m refer to the nature of the resonant mode. Lastly, the background process, or direct pathway, matrix C is modeled with the real parameters $r_{d}$ and $t_{d}=\sqrt{1-r_{d}^{2}}$ for the modulus of the reflection and transmission coefficients, respectively, and ϕ for the global phase, which depends on the reference plane used [55].

With the knowledge of the parameters defined previously (eigenfrequencies of the unperturbed modes, decay rates, coupling strength, and reflection coefficient of the background process), the scattering parameters of the metasurface can be calculated by solving Equation (4), assuming an $e^{j\omega t}$ time dependence, where ω is the angular frequency. The results for the scattering coefficients are given as follows:

(7) $$r^{\pm }\left(\omega \right)=e^{j\phi }\left(r_{d}-\frac{\gamma_{m}A_{p}\left(\omega \right)e^{-j\cos^{-1}\left(r_{d}\right)}+\gamma_{p}A_{m}\left(\omega \right)e^{j\cos^{-1}\left(r_{d}\right)}\pm 2jg\sqrt{\gamma_{p}\gamma_{m}}}{A_{p}\left(\omega \right)A_{m}\left(\omega \right)+g^{2}}\right),$$

(8) $$t\left(\omega \right)=e^{j\phi }\left(jt_{d}+\frac{\gamma_{m}A_{p}\left(\omega \right)e^{-j\cos^{-1}\left(r_{d}\right)}-\gamma_{p}A_{m}\left(\omega \right)e^{j\cos^{-1}\left(r_{d}\right)}}{A_{p}\left(\omega \right)A_{m}\left(\omega \right)+g^{2}}\right),$$

with functions of the angular frequency defined as $A_{p}\left(\omega \right)=j\left(\omega -\omega_{p}\right)+\gamma_{p}$ for the electric resonance, $A_{m}\left(\omega \right)=j\left(\omega -\omega_{m}\right)+\gamma_{m}$ for the magnetic resonance, t for the transmission coefficient, $r^{+}$ for the reflection coefficient for a plane wave with $k_{z}>0$, and $r^{-}$ for the reflection coefficient for a plane wave with $k_{z}<0$. Because of the coupling between resonances, the scattering matrix of the process becomes as follows:

(9) $$|s_{-}\rangle =\left(\begin{array}{cc} r^{-} & t\\ t & r^{+} \end{array}\right)|s_{+}\rangle ,$$

with $\left|r^{+}|=|r^{-}\right|$.

To check the validity of the TCMT model, we perform full‐wave calculations using the structure presented in the previous section and fit the parameters to check the role that they play. The simulated structure, which has $C_{4v}$ symmetry, shares the same form of the S matrix. The reflectance, $R=|r^{\pm }{|}^{2}$, for several values of $\sigma_{p}$, is shown in Figure 2b. The obtained values via FEM simulations, performed using the frequency domain solver in the Wave Optics package in COMSOL Multiphysics, are plotted as squares, whereas the TCMT fit is plotted as a solid line, showing good agreement. Additionally, the retrieved values for the eigenfrequencies from the fit are shown in Figure 2c and for the coupling strength in Figure 2d.

When mirror symmetry is present, $\sigma_{p}=0$, the structure is in a Huygens' configuration, $\omega_{p}≃\omega_{m}$. A pair of even and odd resonances, in this case, electric and magnetic dipole q‐BIC resonances, overlap, resulting in transmission close to one. When out‐of‐plane symmetry is broken, electromagnetic coupling is enabled, and the modes hybridize. The coupling parameter, g, effectively models this electromagnetic omega coupling or, equivalently, the hybridization of the modes. Up to a point, increasing the diameter of the mirror‐breaking perturbation enhances the mode overlap, thereby increasing the coupling strength between the resonances. In the scattering parameters, this effect is translated into an increase in reflectance. However, in detail, two distinct effects are taking place. Firstly, perforating the top of the structure results in a larger shift in frequency for the unperturbed magnetic dipole resonance than for the unperturbed electric dipole resonance. The unperturbed magnetic dipole resonance is a product of the circulation of the electric displacement field, which is highly dependent on the height of the resonator. This is shown in Figure 2c for the values of $\omega_{p}$ and $\omega_{m}$. Secondly, in Figure 2d, it can be seen how the coupling strength increases with the value of $\sigma_{p}$ until it reaches its maximum near $\sigma_{p}=\sqrt{2}/2≃0.707$, and then decreases. This can be explained as for $\sigma_{p}=\sqrt{2}/2$, the area of the perforation is exactly half of the area of the cross‐section of the cylinder.

Indeed, with this formulation of the TCMT model, an interesting property of coupled systems arises. The new eigenfrequencies of the perturbed coupled system, corresponding to the hybridized modes, can be calculated in terms of the eigenfrequencies of the uncoupled (unperturbed) system and the coupling strength; see Supporting Information S1: Section 3. The real parts of the eigenfrequencies of the coupled system (for resonances with equal decay rates) are given as follows:

(10) $$\omega_{\pm }=\left(\frac{\omega_{p}+\omega_{m}}{2}\right)\pm \sqrt{{\left(\frac{\omega_{p}-\omega_{m}}{2}\right)}^{2}+g^{2}},$$

where $\omega_{+}$ and $\omega_{-}$ are the high‐energy hybrid mode (HEHM) and the low‐energy hybrid mode (LEHM) resonance angular frequencies, respectively. This phenomenon is known to occur in two‐level systems, between excitonic resonances and optical resonances [20, 21, 22, 23, 24], as well as between optical modes [17, 25, 26, 33]. As g increases, the two branches separate further from each other, and an anticrossing behavior appears. This anticrossing behavior is usually referred to as Rabi splitting due to its similarity to the strong interaction between two quantum states [56, 57]. In Figure 2c, the two branches are plotted for completeness.

Figure 2d shows the obtained values for the coupling strength in terms of $\sigma_{p}$ and the value $g_{c}=\sqrt{\gamma_{p}\gamma_{m}}$. For the critical value of $g_{c}$, for the specific case of $\omega_{p}=\omega_{m}=\omega_{0}$, the polarizabilities correspond to a balanced particle case [58]; that is, at $\omega =\omega_{0}$, $\alpha_{xx}^{ee}=\alpha_{yy}^{mm}=\alpha_{xy}^{em}$. In Figure 2c, it is shown that for values greater than $\sigma_{p}=0.3$, the coupling strength exceeds this critical value. Additionally, in the literature, it is possible to find the value defined to discern between a weak coupling regime and a strong coupling when $\omega_{p}=\omega_{m}$ as the sum of the decay rates (widths of the resonances) divided by two [59, 60]. This value is chosen because it is similar to the Rayleigh criterion for angular resolution of optical systems. It is considered that such a coupling strength separates the branches sufficiently to be differentiated, forming an anticrossing pattern that allows them to be well distinguished. Using that condition, $|g|>\sqrt{\left(\gamma_{p}^{2}+\gamma_{m}^{2}\right)/2}=g_{\text{strong}}$ is obtained as the strong coupling condition. Detailed derivations can be found in Supporting Information S1: Section 3.

To further demonstrate that the TCMT explains the physics behind the structure, Figure 2e shows the real part of the collective polarizabilities as functions of the TCMT parameters, compared to the values obtained from the FEM simulations. These quantities, which depend on the real and imaginary parts of the scattering coefficients, show good agreement between the simulations and the TCMT. The discrepancy is present due to the quality factor of the resonances, which is in the order of the hundreds; TCMT works better for higher quality factors. Furthermore, the real background process is not constant.

The expressions for the collective polarizabilities in terms of scattering parameters can be written as follows [40]:

(11) $${\hat{\alpha }}_{xx}^{ee}={\hat{\alpha }}_{yy}^{ee}=j\frac{S}{2k}\left(r^{+}-r^{-}+2t-2\right),$$

(12) $${\hat{\alpha }}_{xx}^{mm}={\hat{\alpha }}_{yy}^{mm}=j\frac{S}{2k}\left(2t-2-r^{+}-r^{-}\right),$$

(13) $${\hat{\alpha }}_{xy}^{em}=-{\hat{\alpha }}_{yx}^{em}=-{\hat{\alpha }}_{yx}^{me}={\hat{\alpha }}_{xy}^{me}=-j\frac{S}{2k}\left(r^{+}-r^{-}\right),$$

where S is the area of the periodic lattice cell. Taking into account the results of the TCMT, Equations (7) and (8), these quantities can be written in the following form:

(14) $${\hat{\alpha }}_{xx}^{ee}=\frac{je^{j\phi }S}{k}\left(jt_{d}+r_{d}-2\frac{\gamma_{p}A_{m}\left(\omega \right)e^{j\cos^{-1}\left(r_{d}\right)}}{\left(A_{p}\left(\omega \right)A_{m}\left(\omega \right)+g^{2}\right)}\right)-jS/k,$$

(15) $${\hat{\alpha }}_{yy}^{mm}=\frac{je^{j\phi }S}{k}\left(jt_{d}-r_{d}+2\frac{\gamma_{m}A_{p}\left(\omega \right)e^{-j\cos^{-1}\left(r_{d}\right)}}{A_{p}\left(\omega \right)A_{m}\left(\omega \right)+g^{2}}\right)-jS/k,$$

(16) $${\hat{\alpha }}_{xy}^{em}=-\frac{2e^{j\phi }Sg\sqrt{\gamma_{p}\gamma_{m}}}{\left(A_{p}\left(\omega \right)A_{m}\left(\omega \right)+g^{2}\right)k},$$

where it can be seen how, in the first‐order approximation, ${\hat{\alpha }}_{xy}^{em}$ is linearly proportional to g, whereas ${\hat{\alpha }}_{xx}^{ee}$ and ${\hat{\alpha }}_{yy}^{mm}$ are not affected.

In terms of the coupling strength, there are three cases predicted by this TCMT model that are of special interest. In Figure 3, the three scenarios are shown. All the plots present the crossing between the electric and magnetic dipole modes for different coupling scenarios and for a background direct process with $r_{d}=0$. On the upper row (Figure 3a–c), the TCMT predictions are displayed for two resonances with decay rates set at $\gamma_{m}=2\gamma_{p}$ and $\omega_{m}$ kept constant. The horizontal axis shows the distance between the uncoupled eigenfrequencies, defined as $\Delta \omega =\omega_{m}-\omega_{p}$. In a real metasurface, the distance between resonances can be tuned by changing some geometrical parameter, the refractive index of the structure, or its surroundings. The vertical axis shows the angular frequency centered at $\omega_{m}$. Figure 3a shows the case in which no coupling is allowed, g=0, and for this case, Huygens' condition is met when the resonance lines cross each other. It can be seen that, at the point of crossing, the reflectance is zero and the transmittance is one, as energy conservation requires. In Figure 3b, the condition $g=g_{c}=\sqrt{\gamma_{m}\gamma_{e}}$ ensures that, at the crossing point, the balanced particle condition is fulfilled [39, 58], $r^{+}=-r^{-}=1$ and $\alpha_{xx}^{ee}=\alpha_{xy}^{em}=\alpha_{yy}^{mm}$. Lastly, Figure 3c shows the prediction in the case where $g=4g_{c}=4\sqrt{2}\gamma_{p}>g_{\text{strong}}$. It is clear how the two branches, $\omega_{+}$ and $\omega_{-}$, plotted as dashed lines, show an anticrossing behavior. On the lower row (Figure 3d–f), a similar analysis is done with the asymmetric structure presented in Section 2. The three aforementioned scenarios are replicated using the periodicity of the structure presented in the previous section, P, as the tuning parameter. Under the change of P, the electric dipole resonance shifts in eigenfrequency faster than its magnetic counterpart, making the crossing between them possible. In Figure 3d, Huygens' case is portrayed for the case in which $\sigma_{p}=0$ and, therefore, no coupling is allowed. In Figure 3e, $\sigma_{p}=0.12$ breaks out‐of‐plane mirror symmetry, allowing the coupling between resonances. However, the coupling is not sufficient to clearly distinguish between branches. The coupling in this case is close to the balanced particle case. Figure 3f shows the results for $\sigma_{p}=0.5$, where strong coupling is taking place and both branches separate significantly, producing a clear anticrossing.

**FIGURE 3:**
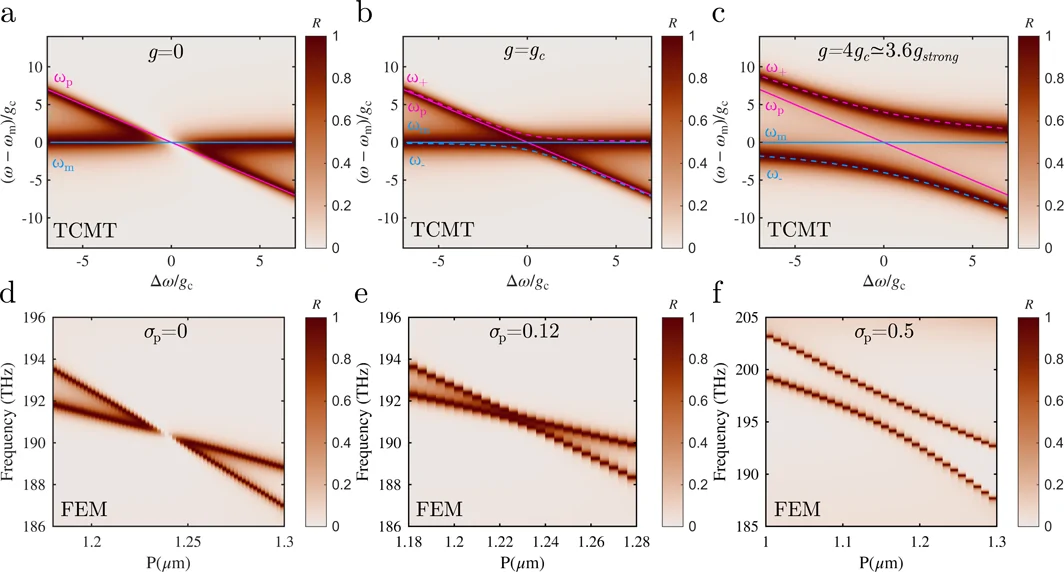
Scattering properties of resonance crossings in off‐diagonal chiral‐type bianisotropic metasurfaces for different coupling scenarios. Upper row: TCMT results have decay rates chosen as $\gamma_{m}=2\gamma_{p}$, with constant background process $r_{d}=0$. The horizontal axis is normalized using $\Delta \omega =\omega_{m}-\omega_{p}$. (a) g=0, no coupling between electric dipole resonance and magnetic dipole resonance. At Δω=0, a Huygens‐like pair is created, achieving R=0 and transmittance T=1. (b) $g=g_{c}$, at the crossing point, the balanced particle is achieved with full reflection. (c) Strong coupling regime, $g=4g_{c}>g_{\text{strong}}$. At Δω=0, a clear anticrossing can be observed. The Rabi‐like splitting can be controlled with the perturbation, as shown in Figure 2. Lower row: FEM results for the crossing controlled by the change of P. (d) Huygens' case with $\sigma_{p}=0$ (e) Balanced particle case with $\sigma_{p}=0.12$, $g\approx g_{c}$. (f) Strong coupling regime with $\sigma_{p}=0.5$ and $g>g_{\text{strong}}$. The geometrical parameters of the metasurface are D=530 nm, Δ=40 nm, h=263 nm, and $h_{p}=20$ nm.

## 4 Dual‐Band Asymmetric Absorption Mediated by Strongly Coupled Modes

In this section, building upon the preceding discussion, we present a general theoretical framework for the design of reciprocal bianisotropic metasurfaces that achieve a maximum difference in directional absorption at optical frequencies. All discussion up to this point has assumed a passive, lossless character for the materials that comprise the structure. However, introducing losses to the broken $\sigma_{h}$ structure opens up the opportunity to have $|r^{-}{|}^{2}\neq |r^{+}{|}^{2}$ [61]. Indeed, with the addition of losses, asymmetric absorption is possible as $\Delta A=A^{+}-A^{-}=|r^{-}{|}^{2}-|r^{+}{|}^{2}$, where $A^{+}$ is the absorption calculated for a wave with $k_{z}>0$ and $A^{-}$ is the absorption calculated for a wave with $k_{z}<0$. Losses can be added into the system as a perturbation when γ≪ω, separating $\gamma_{i}\to \gamma_{i}^{r}+\gamma_{i}^{l}$, where $\gamma_{i}^{r}$ is the radiative part of the decay rate and $\gamma_{i}^{l}$ is the part related to intrinsic losses of the materials [62]. Detailed discussion is presented in Supporting Information S1: Section 4.

For the simplest case in which $\omega_{p}=\omega_{m}=\omega_{0}$ and $\gamma =\gamma_{p}^{r}=\gamma_{p}^{l}=\gamma_{m}^{r}=\gamma_{m}^{l}$, the difference in absorption between sides, evaluated at the LEHM and HEHM frequencies, takes the following form:

(17) $$\Delta A{|}_{\omega =\omega_{\pm }}=\pm sgn\left(g\right)\frac{r_{d}\;g^{2}}{\gamma^{2}+g^{2}}.$$

where sgn() is the sign function. Additionally, with the above‐mentioned restrictions, Equation (10) can now be written as $\omega_{\pm }=\omega_{0}\pm |g|$. Equation (17) shows that asymmetric absorption in the system is bounded by the reflection coefficient of the direct process. This is a similar result to the reported findings in Ref. [55], where the background process bounded the asymmetric decay of a single‐resonance system. In addition, following Equation (17), it can be seen that, in order to maximize ΔA at the resonant frequency of the hybrid mode, it is necessary to have a background process with a high reflection coefficient. Maximum asymmetric directional absorption using strongly coupled resonances is bounded by the reflection coefficient of the background process $r_{d}$. Furthermore, due to the property of Equation (7), $r^{+}\left(-g\right)=r^{-}\left(g\right)$, Equation (17) is multiplied by a sign function that takes g as its argument.

To further elucidate the results, we show in Figure 4a the dependence of the absorption difference with the coupling coefficient for the case in which $\omega_{m}=\omega_{p}=\omega_{0}$, r=1, and $\gamma =\gamma_{p}^{r}=\gamma_{p}^{l}=\gamma_{m}^{r}=\gamma_{m}^{l}$, which fulfills the usual critical coupling condition [63], but it is not enough in this coupled system to achieve full absorption; further discussion can be found in Supporting Information S1: Section 4. The axes are normalized using $g_{c}=\sqrt{\gamma_{p}^{r}\gamma_{m}^{r}}$. It can be seen how, for each branch, $\omega_{+}$ and $\omega_{-}$, the difference in absorption increases with larger values of g. Interestingly, at a given value of g, in the frequency spectrum around $\omega_{0}$, two distinct bands arise. At lower frequencies, one side of the structure has almost full absorption around $\omega \approx \omega_{-}$, whereas the other has full reflection. At higher frequencies, $\omega \approx \omega_{+}$, the responses are interchanged. It is worth noting that the structure is reciprocal; therefore, for ΔA=±1, the transmission vanishes, that is, $|t{|}^{2}=0$.

**FIGURE 4:**
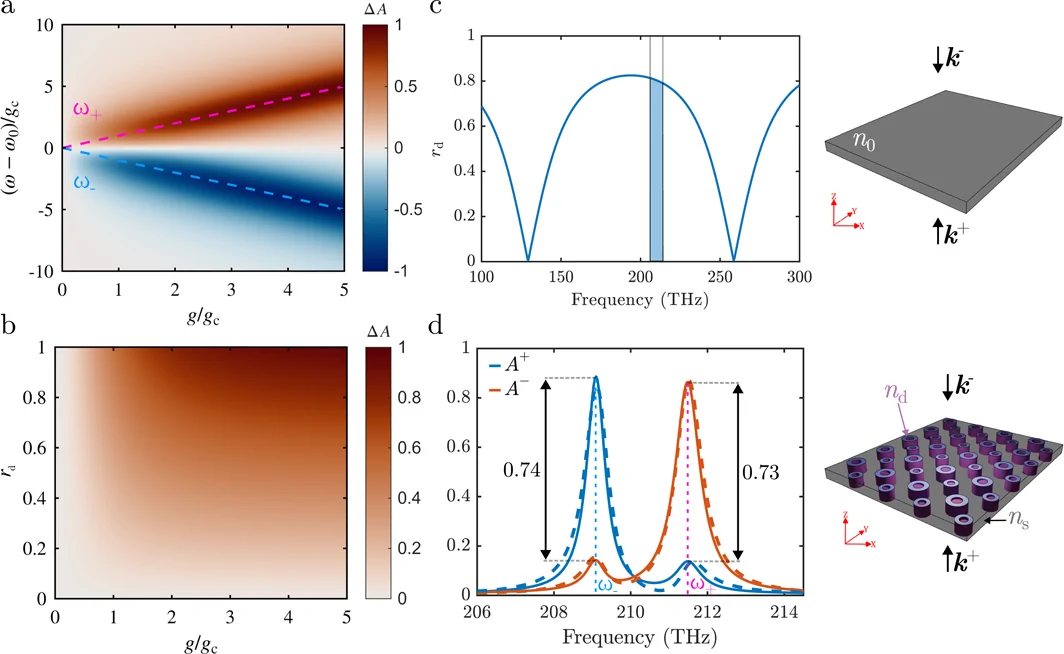
Dual‐band absorption in strongly coupled metasurfaces with off‐diagonal chirality or omega‐type bianisotropy. (a) Case in which $\omega_{p}=\omega_{m}=\omega_{0}$, $\gamma =\gamma_{p}^{r}=\gamma_{p}^{l}=\gamma_{m}^{r}=\gamma_{m}^{l}$, and r=1. The difference in absorption grows with the coupling strength for each branch of the hybrid modes. (b) Absorption difference evaluated at $\omega_{+}$ for $\omega_{p}=\omega_{m}=\omega_{0}$, $\gamma =\gamma_{p}^{r}=\gamma_{p}^{l}=\gamma_{m}^{r}=\gamma_{m}^{l}$ in terms of absolute value of the reflection coefficient for the background process and coupling strength. (c) Reflection from a Fabry–Pérot slab of dielectric of height h=360 nm and effective refractive index $n_{0}=3.22$. (d) Calculated absorption via FEM simulations for the metasurface embedded in a dielectric slab. Solid lines represent FEM results, whereas the TCMT fit is represented by dashed lines. FEM geometrical parameters and material properties: P=510 nm, Δ=70 nm, $h_{p}=40$ nm, $\sigma_{p}=0.5$, h=360 nm, D=280 nm, $n_{s}=2.5$, $n_{d}=4-jk$, and k=0.005 as the imaginary part of the refractive index of the nanodisks.

Figure 4b shows the dependence of ΔA on the coupling strength and reflection coefficient, as Equation (17) predicts. Following the recipe provided by the equation, we demonstrate how to design a metasurface with a similar scattering response, that is, with a maximum directional absorption difference. The main idea is to embed the above‐shown metasurface, with broken out‐of‐plane symmetry, in a dielectric slab. The slab emulates a direct scattering process with high reflectivity, specifically a Fabry–Pérot resonator. We show in Figure 4c the broadband response of a slab with effective refractive index $n_{0}=\left(n_{s}V_{\text{Slab}}^{fr}+n_{d}V_{\text{Disks}}^{fr}\right)$, where $n_{s}$ is the refractive index of the host slab, similar to the refractive index of ${TiO}_{2}$, and $n_{d}$ is the refractive index of the embedded structure similar to the refractive index of germanium, $V_{\text{Slab}}^{\mathrm{fr}}$ and $V_{\text{Disks}}^{fr}$ are the volume fractions occupied by the slab structure and the disks, respectively. This procedure is similar to that used in Ref. [51]. Because of the fact that the decay rates of the Fabry–Pérot resonances of the slab are significantly larger than those of the resonances held by the metasurface, it can be regarded as a constant direct pathway. We choose a thickness such that the modulus of the reflectivity is high near 210 THz, $r_{d}≃0.8$, as shown in the blue region of Figure 4c. Afterward, the metasurface geometrical parameters are chosen such that $\omega_{m}=\omega_{p}$. The drilled hole on top of the nanodisks enables strong mode coupling between the modes and bianisotropic omega behavior.

The result from the FEM simulations is shown in Figure 4d, where the dual‐band behavior can be clearly seen. To bring the electric and magnetic resonances together in this embedded metasurface, large changes to the geometrical parameters had to be made in comparison with the structures shown in the previous sections. Furthermore, $\sigma_{p}$ was used to tune the coupling, and additionally, $h_{p}$ was also used to tune electromagnetic coupling, as shown in Ref. [38]. A symmetry in the absorption spectra with respect to 210 THz can be seen, as $\gamma_{p}$ is similar to $\gamma_{m}$. Fitted results using the TCMT are plotted as dashed lines, showing good agreement. With this straightforward approach, and without any optimization, more than a 73% difference in directional absorption is reached at the resonant frequencies of the HEHM and the LEHM. The calculated coupling strength is equal to g=−1.21 THz, which is 5.72 times larger in magnitude than $|g_{\text{strong}}|=0.21$ THz; therefore, the modes are strongly coupled. Nevertheless, the modes do not have the exact same scattering decay rates, making the response not symmetrical around 210 THz. However, a rough estimation of the absorption difference limit can still be performed using Equation (17). Plugging in g, γ=0.21 THz, and the fitted reflectivity for the background, $r_{d}\approx 0.8$, the result is that $\Delta A{|}_{\omega =\omega_{\pm }}\approx \mp 0.78$, which is a value near the one obtained with FEM simulations at $\omega_{+}$ and $\omega_{-}$ for the difference in directional absorption. The presented design exemplifies the practical implementation of the model, serving as a theoretical approach for designing metasurface‐based devices that require directional control of light absorption. To maximize ΔA, multiple strategies could be further implemented: improving the background to have higher reflectivity, using higher‐quality‐factor resonances, or further increasing g by using a deeper perforation on top of the cylinders.

### 4.1 Substrate‐Induced Coupling of Modes

Previously, we have used a geometrical perturbation, a drilled hole, to break mirror symmetry in the nanodisk lattice and control the electromagnetic coupling. However, a more suitable approach compatible with common nanofabrication techniques would be to break mirror symmetry solely using the substrate. Omega bianisotropy induced by a substrate has been studied in plasmonic [31] and dielectric structures [64, 65]. However, in those studies, BICs were not explored. Additionally, maximum diagonal bianisotropy, chirality, has recently been shown to be possible with the use of a substrate in Ref. [66].

In this section, we show how, without using holes in the nanodisks $\left(\sigma_{p}=0\right)$ and using a substrate with refractive index $n_{subs}=1.45$, the same dual‐band absorption effect as that shown in Figure 4 can be achieved. In this configuration, the coupling between the two resonances is enabled by the substrate. This presents a couple of drawbacks, as the addition of a substrate makes the resonances shift to lower frequencies and have higher leakage, that is, lower quality factors and larger decay rates. Furthermore, the control of the coupling parameter can only be achieved by changing the refractive index of the substrate or by changing the geometry of the whole structure to make the modes more or less electromagnetically confined and, therefore, control the amount of near fields that interact with the substrate, which leads to the coupling and hybridization of eigenmodes.

We use the same material parameters for the disks and the slab in which they are embedded as shown in Figure 4, that is, $n_{d}=4$ and $n_{s}=2.5$, respectively. A dielectric substrate with refractive index $n_{subs}=1.45$, a refractive index similar to that of ${\text{SiO}}_{2}$ at near‐infrared frequencies, is added below the structure. The calculated absolute value of reflectivity of the slab, with effective refractive index $n_{0}\approx 3.15$, lying above the substrate is shown in Figure 5a. Several interesting features arise in comparison with Figure 4c. The background reflectivity now has an offset of around $\min\left(r_{d}\right)=0.183$. This offset can be exactly obtained by calculating the reflectivity using the Fresnel equations for normal incidence at an air–silica interface. Furthermore, the reflectivity now reaches a lower maximum, and its resonant features are smoothed. At the central design frequency for the device, $r_{d}\left(203\;THz\right)=0.74$.

**FIGURE 5:**
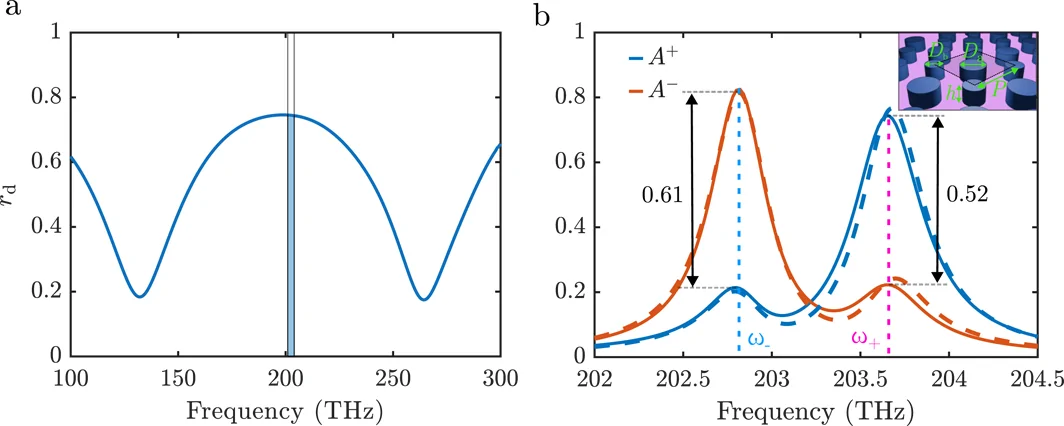
Substrate‐induced dual‐band absorption in strongly coupled omega metasurfaces. (a) Background reflection calculated for the dielectric slab of height h=360 nm, $n_{0}=3.15$, and a semi‐infinite substrate with refractive index $n_{subs}=1.45$. The frequency zone of interest is shaded in blue. (b) FEM‐simulated structure showing directional absorption contrast for a mirror‐symmetric metasurface embedded on a dielectric with refractive index $n_{s}$ on top of a substrate with $n_{subs}=1.45$. Geometrical parameters and material properties: P=535 nm, Δ=50 nm, h=360 nm, D=280 nm, $n_{s}=2.5$, $n_{d}=4-jk$, and k=0.003.

In Figure 5b, the results for the FEM‐simulated metasurface positioned on top of the dielectric substrate are shown. The dual‐band response is exhibited by the metasurface with a contrast higher than 50%. Interestingly, the position of the mirror‐symmetry‐breaking element (substrate) is now on the opposite side of the metasurface compared to the scenario in Figure 4d. Indeed, in the previous case, the holes were made on top of the metasurface. This is reflected in the sign of the coupling parameter, which, for the presented figure, reaches a value of g=0.44 THz. Following Equation (17), as the sign changes, the bands interchange positions compared to Figure 4d. This exemplifies the fact that, by controlling on which side the perturbation is made, the sign of ΔA can also be controlled at the resonant frequencies of the hybridized modes. In contrast with the previous design, the periodicity of the structure had to be changed to bring $\omega_{p}$ and $\omega_{m}$ close to each other after the addition of the substrate. Parameter Δ was decreased to achieve higher quality factors for obtaining good contrast in directional absorption. Additionally, k, the dielectric loss, had to be adjusted to balance decay rates due to intrinsic losses and decay rates due to radiative losses. All the optimized parameters can be found in the caption of Figure 5.

## 5 Conclusions

In this work, we have developed a comprehensive and physically intuitive framework to describe omega‐type bianisotropy in dielectric metasurfaces operating under high‐quality resonances. By using a temporal coupled‐mode theory (TCMT) model, we have shown how controlled out‐of‐plane symmetry breaking activates electromagnetic coupling between electric and magnetic dipolar resonances of opposite parity, leading to their hybridization and to the emergence of a Rabi‐like splitting characteristic of the strong coupling regime. The coupling can be controlled by the amount of out‐of‐plane symmetry breaking introduced and can lead to a very strong bianisotropic response in a deeply subwavelength structure. In fact, the structure shown is a reciprocal analog of a pure moving medium, as shown in Ref. [44]. In contrast to many studies in the literature, the anticrossing behavior of the system can be observed directly from the parameters calculated via TCMT rather than from the solutions of an effective Hamiltonian and subsequent Fano fitting.

Furthermore, this study presents a comprehensive theoretical model for designing optical reciprocal metasurfaces with maximum asymmetric directional absorption. We have demonstrated that, when intrinsic losses are introduced, these strongly coupled hybrid modes produce a dual‐band asymmetric absorption response under counterpropagating illumination. A realization of such an optical response can be achieved by embedding the metasurface in a carefully designed dielectric slab acting as a Fabry–Pérot resonator, which enhances the background reflectivity, allowing the directional absorption contrast to exceed 70% in two distinct spectral bands. Furthermore, the same concept is demonstrated for a mirror‐symmetric array of nanodisks positioned on a dielectric substrate: a geometry compatible with available nanofabrication techniques. This behavior represents an efficient and fully reciprocal approach to implementing maximum direction‐dependent light dissipation in passive optical systems.

## Author Contributions

**L. M. Manez‐Espina:** conceptualization, investigation, writing – original draft, formal analysis, validation, visualization, writing – review and editing, data curation. **B. Amrahi:** writing – original draft, writing – review and editing, validation, visualization, investigation. **V.S. Asadchy:** writing – original draft, investigation, validation, writing – review and editing, supervision, conceptualization, funding acquisition, visualization. **A. Diaz‐Rubio:** writing – original draft, funding acquisition, conceptualization, investigation, visualization, writing – review and editing, supervision, validation.

## Funding

This work was supported by Agencia Estatal de Investigación (Grants PID2024‐162261NB‐I00 and CNS2024‐154715), Generalitat Valenciana under the PROMETEO Program (CIPROM/2022/14), Universitat Politècnica de València (Grant PAID‐01‐23), and the Academy of Finland (Grants 356797, 320167, and 368652)

## Supporting information


Supporting Information S1

## Data Availability

The data that support the findings of this study are available from the corresponding author upon reasonable request.
