# Supplementary material for: Strong Mode Coupling and Maximum Directional Absorption in Reciprocal Bianisotropic Metasurfaces
Source: Nanophotonics. 2026 Jun 16;15(12):e70145. doi: 10.1002/nap2.70145 (PMC13642634; doi:10.1002/nap2.70145)
Supplement: Supplementary file 1 [file NAP2-15-e70145-s001.pdf]

Supplementary Material: Strong Mode Coupling and Maximum Directional Absorption in Reciprocal Bianisotropic Metasurfaces

L.M. Máñez-Espina*[1], B. Amrahi[2], V.S. Asadchy[2], and A. Díaz-Rubio[1]

[1]Nanophotonics Technology Center, Universitat Politècnica de València, València 46022, Spain.
[2]Department of Electronics and Nanoengineering, Aalto University, Espoo, 02150, Finland.
*lmmaeesp@ntc.upv.es; bahman.amrahi@aalto.fi; viktar.asadchy@aalto.fi; andiaru@upv.es

1 Coupling Integrals and Group Symmetry Origins of q-BIC Modes

The q-BIC modes in the case of the mirror symmetric structure do belong to the two-dimensional irreducible representations (the eigenmodes come in degenerated pairs) of the $D_{4\mathrm{h}}$ group: the magnetic dipole group belongs to the E_g representation that transforms antisymmetrically upon σ_h symmetry operation (mirror symmetry with respect to the $x - y$ plane) and the electric dipole resonance belongs to the E_u representation that transforms symmetrically upon σ_h, see Table S1 of this Supplementary. However, when out-of-plane mirror symmetry is broken, the symmetry group is reduced from $D_{4\mathrm{h}}$ to $C_{4\mathrm{v}}$, and the irreducible representations of both electric and magnetic dipole resonances coalesce into the E two-dimensional representation of $C_{4\mathrm{v}}$, see Table S2 of this Supplementary. This symmetry reduction enables bianisotropic electromagnetic coupling. Indeed, under $D_{4\mathrm{h}}$ symmetry, if $\mathbf{E_p}$ and $\mathbf{E_m}$ denote the electric field distributions of the electric and magnetic dipole resonances, respectively, their parity with respect to z inversion satisfies $\mathbf{E_p}(z) = \mathbf{E_p}(-z)$ and $\mathbf{E_m}(z) = -\mathbf{E_m}(-z)$. Consequently, the overlap integral vanishes:

$$\begin{aligned}\psi_{pm} = \int_V \mathbf{E_p}(z) \cdot \mathbf{E_m}(z)^* dV = \int_{V^+} \mathbf{E_p}(z) \cdot \mathbf{E_m}(z)^* dV + \int_{V^-} \mathbf{E_p}(z) \cdot \mathbf{E_m}(z)^* dV = \\ \int_{V^+} \mathbf{E_p}(z) \cdot \mathbf{E_m}(z)^* dV + \int_{V^+} \mathbf{E_p}(-z) \cdot \mathbf{E_m}(-z)^* dV = \\ \int_{V^+} \mathbf{E_p}(z) \cdot \mathbf{E_m}(z)^* dV - \int_{V^+} \mathbf{E_p}(z) \cdot \mathbf{E_m}(z)^* dV = 0,\end{aligned} \tag{1}$$

where "·" is the usual dot product, V is the total volume of integration, V^+ is the upper half space, V^- is the lower half space, and * denotes complex conjugation. When σ_h symmetry is broken, the overlap integral given in Eq. (1) becomes non-zero [1].

D_{4h}	E	$2C_4$	C_2	$2C_2'$	$2C_2''$	i	$2S_4$	σ_h	$2\sigma_v$	$2\sigma_d$
A_{1g}	1	1	1	1	1	1	1	1	1	1
A_{2g}	1	1	1	-1	-1	1	1	1	-1	-1
B_{1g}	1	-1	1	1	-1	1	-1	1	1	-1
B_{2g}	1	-1	1	-1	1	1	-1	1	-1	1
E_g	2	0	-2	0	0	2	0	-2	0	0
A_{1u}	1	1	1	1	1	-1	-1	-1	-1	-1
A_{2u}	1	1	1	-1	-1	-1	-1	-1	1	1
B_{1u}	1	-1	1	1	-1	-1	1	-1	-1	1
B_{2u}	1	-1	1	-1	1	-1	1	-1	1	-1
E_u	2	0	-2	0	0	-2	0	2	0	0

Table S1: Character table of D_{4h}. The table is shown in standard Mulliken representation.

C_{4v}	E	$2C_4$	C_2	$2\sigma_v$	$2\sigma_d$
A_1	1	1	1	1	1
A_2	1	1	1	-1	-1
B_1	1	-1	1	1	-1
B_2	1	-1	1	-1	1
E	2	0	-2	0	0

Table S2: Character table of C_{4v}

2 Quasi-Bound States in the Continuum in the Considered Metasurfaces

The use of q-BIC modes enables the engineering of radiative losses by tuning the perturbation parameter Δ. Furthermore, TCMT is a first-order approximation of Maxwell equations that only works properly when $\gamma << \omega$, i.e., when high-quality-factor resonances are present. For these reasons, q-BICs are the appropriate platform for testing the proposed TCMT model. Here we show how the mirror-symmetric structure shown in Figure 1(c) of the main document supports q-BICs. These resonances follow the known scaling rule for symmetry-protected BICs, $Q_{\text{qBIC}} = B/\Delta^2$ [2, 3], being Q_{qBIC} the quality factor of the q-BIC mode, B a constant that depends on the geometry, and Δ the perturbation parameter. Figure S1 of this Supplementary Materials shows the dependence of the quality factor on Δ^{-2}. Linear regression proves how the scaling law is followed by the modes used. In this study, complex eigenfrequencies were obtained by FEM simulation using COMSOL Multiphysics, labeled $f = f_{\text{r}} - jf_{\text{i}}$ and used to compute the quality factor as $Q = f_{\text{r}}/(2f_{\text{i}})$.

An important remark to the reader: the results in Figure S1 of this Supplementary Materials do follow a linear function (dashed black line). However, the visual appearance of the plots is not linear, as the used axes are in logarithmic scales distorts the linear trend into a curve. Using linear axis wouldn't allow to see enough points in the study.

The mechanism that brings the dark modes, $\Delta = 0$, to bright modes is extensively studied in [3] and references therein. It takes the name of First Brillouin Zone folding, as changing the diameter of nearest neighbour nanodisks doubles the size of the geometrical lattice and, in the reciprocal space, shrinks the reciprocal lattice by folding the X point into the Γ. Therefore, bringing the dark modes in the X to the Γ point and making coupling with normal illumination possible.

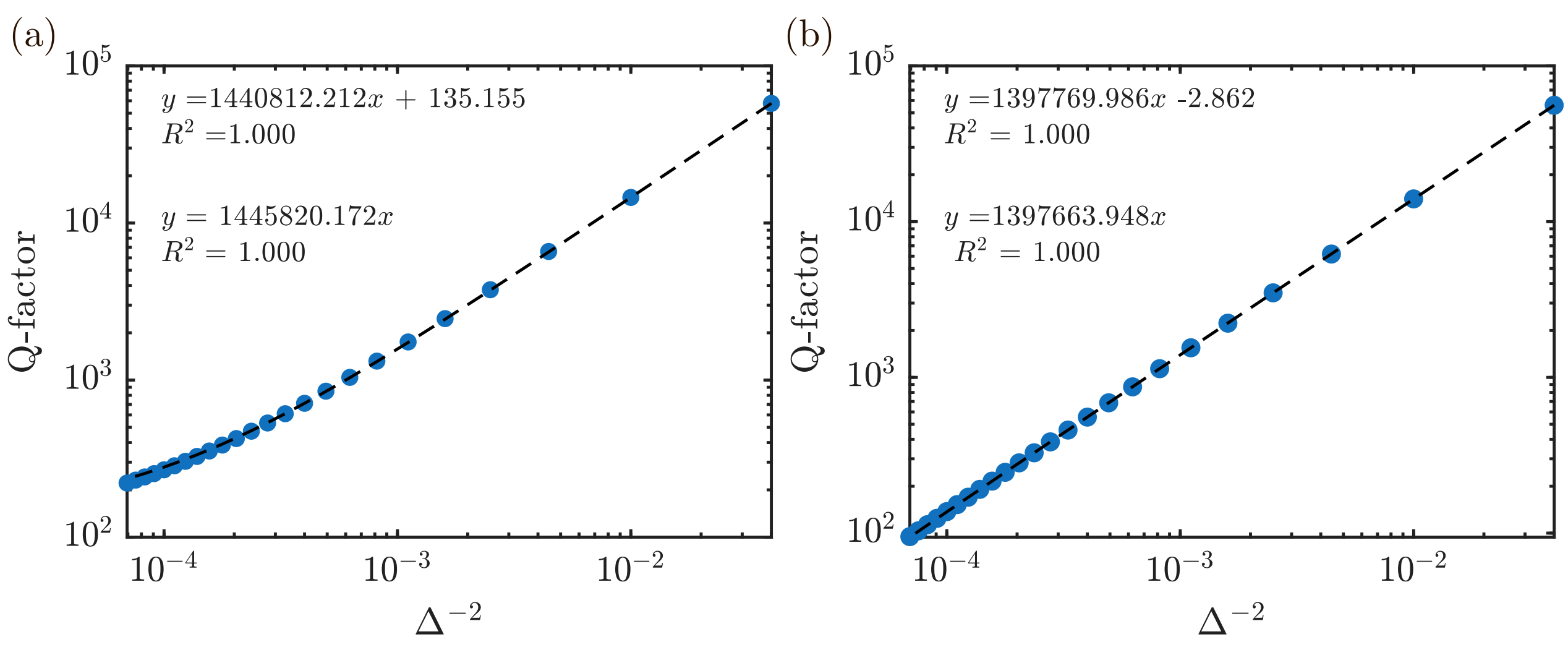

Figure S1: **Analysis of the q-BICs quality factor scaling law.** (a) Shows the dependence of the extracted quality factor for the electric dipole-like resonant mode of the metasurface versus the perturbation Δ. Axes are in logarithmic scale for better visualization. The structure used has the exact dimensions of that shown in Figure 1(c) of the main document: $P = 1000$ nm $D = 600$ nm, $h = 250$ nm, and $n_{\text{d}} = 3.5$. (b) Shows the same study but for the magnetic dipole mode. In both plots, a linear regression has been done, shown as a dashed line. Almost exact agreement is obtained. These results demonstrate that the observed resonances in the metasurface follow the same scaling law $Q \sim \Delta^{-2}$ as conventional quasi-BICs. In the plots, R^2 is the coefficient of determination for the linear regression.

The aforementioned modes are perturbed out-of-plane in Figure 1(d) of the main text to allow electromagnetic

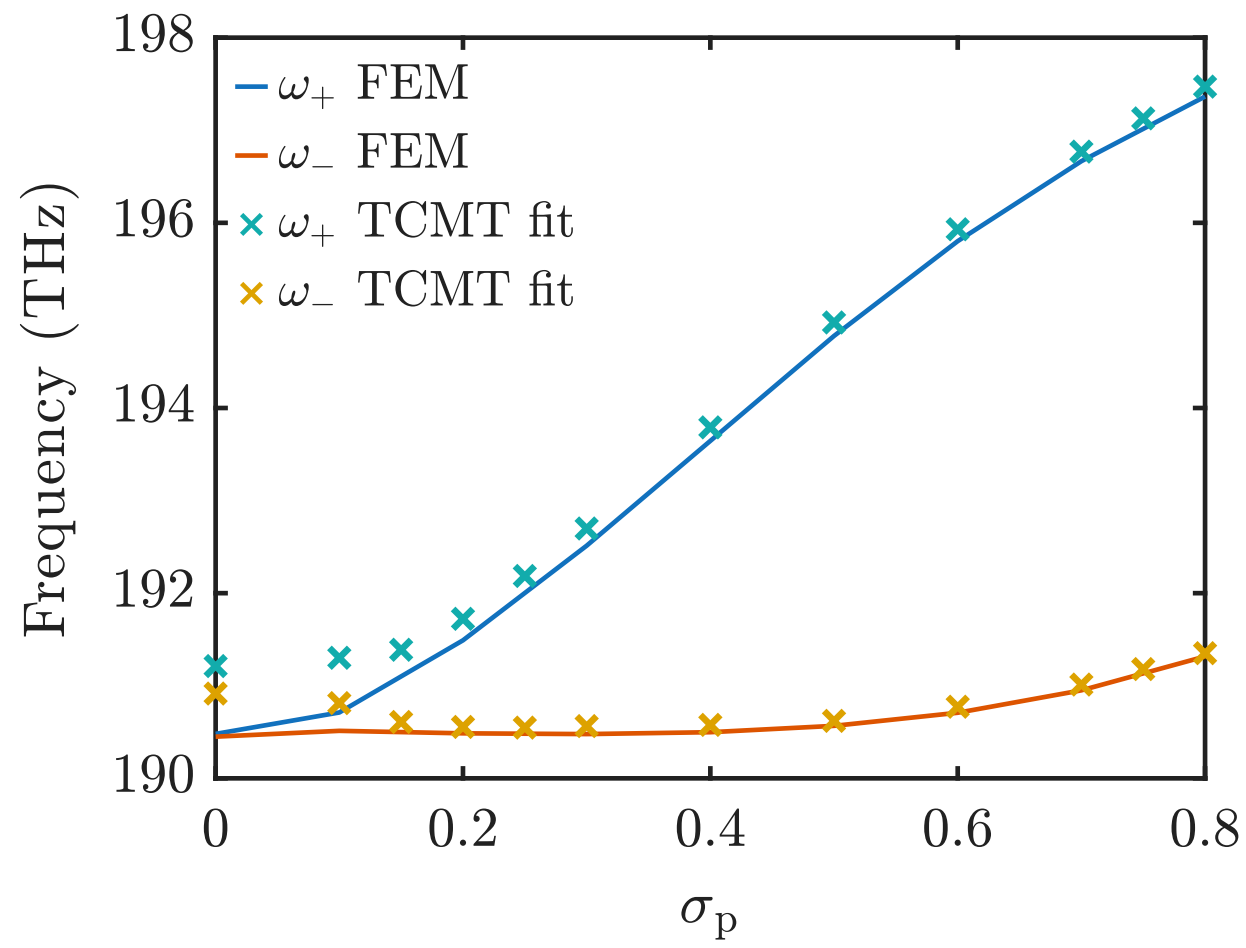

Figure S2: **Eigenvalue comparison between FEM simulations and TCMT fit.** The eigenvalues for the two branches are represented for the metasurfaces shown in Figure 2(b) of the main text. The fitted and calculated values for the eigenvalues show a good fit. The eigenfrequency solver in the Wave Optics module of COMSOL was used.

coupling. Figure 2 (c) of the main text shows the fitted results for the eigenvalues of the branches. Here, in Figure S2 of the Supplementary Materials, we plot the fitted values obtained versus the FEM calculated eigenvalues of the structure, showing good agreement.

3 Strong Coupling Criteria and Balanced Particle

The eigenvalues of the coupled system can be found by solving the equation $(j\Omega - \Gamma - j\omega I)\mathbf{a} = 0$, which encapsulates the dynamics of the amplitudes. Therefore, solving

$$\begin{vmatrix} j(\omega_p - \omega) - \gamma_p & jg \\ jg & j(\omega_m - \omega) - \gamma_m \end{vmatrix} = 0, \tag{2}$$

and calculating the roots of this second-order polynomial, we arrive at

$$\tilde{\omega}_\pm = \left(\frac{\omega_\mathrm{m} + \omega_\mathrm{p}}{2}\right) + j\left(\frac{\gamma_\mathrm{m} + \gamma_\mathrm{p}}{2}\right) \pm \sqrt{g^2 - \left(\frac{j(\omega_\mathrm{m} - \omega_\mathrm{p})}{2} + \frac{\gamma_\mathrm{m} - \gamma_\mathrm{p}}{2}\right)^2}, \tag{3}$$

where complex frequencies $\tilde{\omega}_+$ and $\tilde{\omega}_-$ are the high-energy hybrid mode (HEHM) and the low-energy hybrid mode (LEHM).

Assuming that $\omega_p = \omega_m$, one can define the distance between both branches as

$$\Theta = \tilde{\omega}_+ - \tilde{\omega}_- = 2\sqrt{g^2 - \left(\frac{\gamma_\mathrm{m} - \gamma_\mathrm{p}}{2}\right)^2}. \tag{4}$$

Notice that this is labeled in some studies as $2\hbar\Omega_\mathrm{Rabi}$. With Ω_Rabi corresponding to the vacuum Rabi oscillation frequency.

To be able to differentiate between branches inspecting the scattering amplitude coefficients, some criteria must be given. It is straightforward to check that for $\Re(\Theta) > 0$, g must be equal or greater than $|\gamma_\mathrm{m} - \gamma_\mathrm{p}|/2$. However, with $g = |\gamma_\mathrm{p} - \gamma_\mathrm{m}|/2$, both branches will not be distinguishable. With these considerations in mind, we choose, following the usual procedure, that for the two branches to be properly distinguished, it is sufficient that their separation is greater than the sum of their decay rates. Therefore, we can calculate the criteria for strong coupling as

$$\Theta > \gamma_\mathrm{p} + \gamma_\mathrm{m} \rightarrow g_\mathrm{strong} > \sqrt{(\gamma_\mathrm{m}^2 + \gamma_\mathrm{p}^2)/2} \tag{5}$$

To be precise, both conditions must be fulfilled for strong coupling to be present. However, as for usual structures γ_p and γ_m are different but not extremely separated in value, $g_\mathrm{strong} > \sqrt{(\gamma_\mathrm{m}^2 + \gamma_\mathrm{p}^2)/2}$ is the strongest constraint in our simulations and studies.

Following with the argument, we can rewrite Eq. 3 as

$$\tilde{\omega}_{\pm} = \left(\frac{\omega_{\rm m}+\omega_{\rm p}}{2}\right) + j\left(\frac{\gamma_{\rm m}+\gamma_{\rm p}}{2}\right) \pm \sqrt{g^2 - \left(\frac{j\Delta\omega+\Delta\gamma}{2}\right)^2}, \tag{6}$$

with $\Delta\gamma = \gamma_{\rm m} - \gamma_{\rm p}$ and $\Delta\omega = \omega_{\rm m} - \omega_{\rm p}$. The real part of this equation is

$$\omega_{\pm} = \Re(\tilde{\omega}_{\pm}) = \left(\frac{\omega_{\rm m}+\omega_{\rm p}}{2}\right) \pm \Re\left(\sqrt{g^2 - \left(\frac{j\Delta\omega+\Delta\gamma}{2}\right)^2}\right) \tag{7}$$

For our studies, where we use high-quality resonances, we have that both $\gamma << \omega_p$ and $\gamma << \omega_m$ are met. To see the dynamics of the branches, we can calculate the real part of the complex eigenfrequencies in this approximation, and for a small mismatch in the decay rates, $\Delta\gamma = \gamma_{\rm m} - \gamma_{\rm p} << 1$, as

$$\omega_{\pm} = \Re(\tilde{\omega}_{\pm}) \approx \left(\frac{\omega_{\rm p}+\omega_{\rm m}}{2}\right) \pm \sqrt{\left(\frac{\omega_{\rm p}-\omega_{\rm m}}{2}\right)^2 + g^2}. \tag{8}$$

This equation is shown in the main text as Equation 11, and it is used to illustrate the anticrossing behaviour of this coupled systems.

Additionally, a relevant point in our discussion is that the special case, usually referred to as balanced particle [4], in which $\alpha^{ee} = \alpha^{em} = \alpha^{mm}$ can be achieved when $\omega_p = \omega_m$ for $g_{\rm c} = \sqrt{\gamma_m \gamma_p}$. This balanced particle case, in the absence of material losses, gives reflection coefficients that differ by a phase of π for opposite propagation directions $r^+ = -r^-$.

4 Maximum Directional Absorption Difference

Introducing material losses brings the possibility of changing absorption for different propagation directions. Losses are added to the TCMT as an additional term to the decay rates as done in [5, 6] and other works. Equations 8 and 9 of the main text are the solutions to the dynamical equations with reciprocity and energy conservation constraints. In those equations, all decay rates and exponentials (that depend on $r_{\rm d}$) that are outside the functions, $A_{\rm p}$ and $A_{\rm m}$, are due to reciprocity and energy conservation constraints. Indeed, they are the solutions for the coupling coefficients of matrix D in terms of decay rates and the parameters of the direct channel. Therefore, to introduce losses, it is sufficient, as a first approximation, to expand $A_{\rm p}^{\rm lossy}(\omega) = j(\omega-\omega_{\rm p}) + \gamma_{\rm p}^{\rm r} + \gamma_{\rm p}^{\rm l}$ and $A_{\rm m}^{\rm lossy}(\omega) = j(\omega-\omega_{\rm m}) + \gamma_{\rm m}^{\rm r} + \gamma_{\rm m}^{\rm l}$ and leave all other decay rates γ's as γ^r's. The equations will then acquire the form

$$r^{\pm}(\omega) = e^{j\phi}\left(r_{\rm d} - \frac{\gamma_{\rm m}^{\rm r} A_{\rm p}^{\rm lossy}(\omega) e^{-j\cos^{-1}(r_{\rm d})} + \gamma_{\rm p}^{\rm r} A_{\rm m}^{\rm lossy}(\omega) e^{j\cos^{-1}(r_{\rm d})} \pm 2jg\sqrt{\gamma_{\rm p}^{\rm r}\gamma_{\rm m}^{\rm r}}}{A_{\rm p}^{\rm lossy}(\omega) A_{\rm m}^{\rm lossy}(\omega) + g^2}\right), \tag{9}$$

$$t(\omega) = e^{j\phi}\left(jt_{\rm d} + \frac{\gamma_{\rm m}^{\rm r} A_{\rm p}^{\rm lossy}(\omega) e^{-j\cos^{-1}(r_{\rm d})} - \gamma_{\rm p}^{\rm r} A_{\rm m}^{\rm lossy}(\omega) e^{j\cos^{-1}(r_{\rm d})}}{A_{\rm p}^{\rm lossy}(\omega) A_{\rm m}^{\rm lossy}(\omega) + g^2}\right), \tag{10}$$

with $A_{\rm p}^{\rm lossy}(\omega) = j(\omega-\omega_{\rm p}) + \gamma_{\rm p}^{\rm r} + \gamma_{\rm p}^{\rm l}$ and $A_{\rm m}^{\rm lossy}(\omega) = j(\omega-\omega_{\rm m}) + \gamma_{\rm m}^{\rm r} + \gamma_{\rm m}^{\rm l}$. From this equations, the known as critical coupling condition $\gamma^{\rm r} = \gamma^{\rm l}$ for full absorption also holds in the case in which $\omega_{\rm m} = \omega_{\rm p}$ and $g = 0$ [7]. However, due to the energy exchange enabled by g between resonant states, the critical coupling condition will not yield full absorption. Indeed, it can be calculated that for $\gamma_{\rm m}^{\rm r} = \gamma_{\rm m}^{\rm l} = \gamma_{\rm p}^{\rm r} = \gamma_{\rm p}^{\rm l} = \gamma$ and $\omega_{\rm m} = \omega_{\rm p}$ at ω_+

$$A^{\pm}|_{\omega=\omega_{\pm}} = \frac{\gamma^2 + 0.5g^2(1+r_{\rm d})}{\gamma^2+g^2}, \tag{11}$$

which can only be equal to one if $g = 0$ or if $r_{\rm d} = 1$.

In Figure 4 of the main text, an effective refractive index is given for the simulated slab. Here we give the details of its calculation using the fractions of volume that each structure occupies. The effective refractive index of the dielectric slab used to calculate its Fabry-Perot resonances is defined as

$$n_0 = (n_{\rm s} V_{\rm Slab}^{\rm fr} + n_{\rm d} V_{\rm Disks}^{\rm fr}) \simeq 3.22, \tag{12}$$

where the fractional volumes are given as

$$V_{\text{Disks}}^{\text{fr}} = (\pi(D_a/2)^2 h + \pi(D_b/2)^2 h)/(hP^2), \tag{13}$$

and

$$V_{\text{Slab}}^{\text{fr}} = 1 - V_{\text{Disks}}^{\text{fr}}. \tag{14}$$

5 Substrate Role in Directional Absorption

Using the metasurface presented in section 4 of the main text, and adding a substrate to the structure with $n_{\text{sub}} = 1.4$, the difference in absorption between sides drops due to the lower quality factors of the resonances, see Figure S3 of this Supplementary Materials. The quality factors of the resonances drop slightly due to a lower index contrast between the structure and the substrate that creates leakage. Additionally, due to the high refractive index of the materials used, the resonances shift to lower frequencies. However, the dual-band effect remains strong and noticeable.

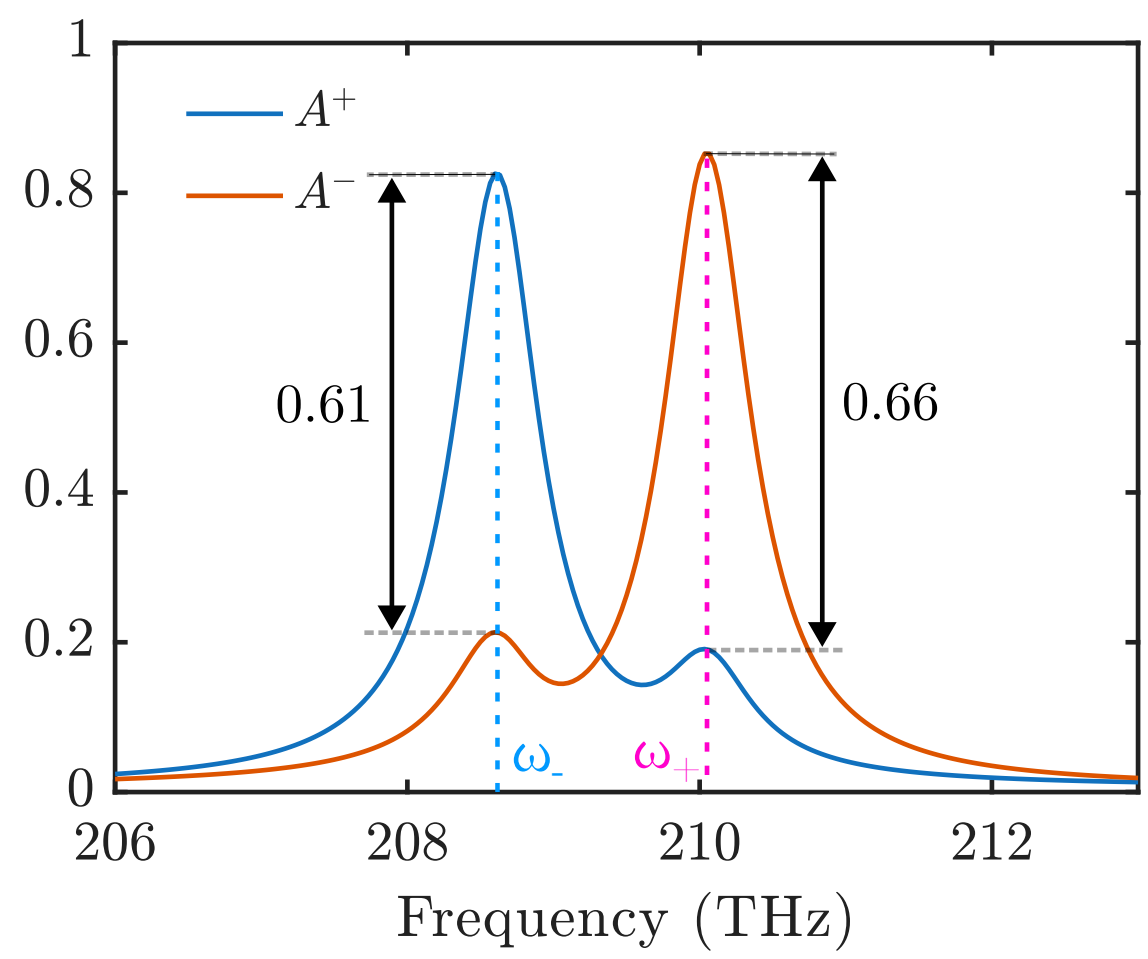

Figure S3: **FEM calculated absorption for the metasurface with broken mirror symmetry located on a substrate.** The metasurface geometrical parameters are shown the same that does of Figure 4 (d): $n_{\text{s}} = 2.5$, $n_{\text{d}} = 4 - jk$, $P = 510$ nm, $\Delta = 70$ nm, $h_{\text{p}} = 40$ nm, $\sigma_{\text{p}} = 0.5$, $h = 360$ nm, $D = 280$ nm, and $k = 0.005$. The substrate used has refractive index $n_{\text{sub}} = 1.4$

.

6 Polarizabilities: Dimensions and Units

In the main text, polarizabilities are defined to have dimensions of volume:

$$[\alpha^{\text{em}}] = [\alpha^{\text{ee}}] = [\alpha^{\text{mm}}] = \text{m}^3 \tag{15}$$

However, in many studies [8, 9], polarizabilities are usually defined with the following units:

$$[\alpha^{\text{em}}] = [\eta_0 \alpha^{\text{ee}}] = [\alpha^{\text{mm}}/\eta_0] = \text{m}^2 \cdot \text{s} \tag{16}$$

The following relation connects both definitions. We use $_{(c)}$ and $_{(a)}$ to denote the current convention and the alternative convention found in the literature.

$$\alpha_{(\text{c})}^{\text{ee}} = \alpha_{(a)}^{\text{ee}}/\varepsilon_0 \tag{17}$$

$$\alpha_{(\text{c})}^{\text{mm}} = \alpha_{(a)}^{\text{mm}}/\mu_0 \tag{18}$$

$$\alpha_{(\text{c})}^{\text{em}} = \alpha_{(a)}^{\text{em}} c = \frac{\alpha_{(a)}^{\text{em}}}{\sqrt{\varepsilon_0 \mu_0}} \tag{19}$$

References

1. Gladyshev, S., Frizyuk, K. & Bogdanov, A. Symmetry analysis and multipole classification of eigenmodes in electromagnetic resonators for engineering their optical properties. *Physical Review B* **102,** 075103 (2020).
2. Koshelev, K., Lepeshov, S., Liu, M., Bogdanov, A. & Kivshar, Y. Asymmetric metasurfaces with high-Q resonances governed by bound states in the continuum. *Physical review letters* **121,** 193903 (2018).
3. Overvig, A. C., Malek, S. C., Carter, M. J., Shrestha, S. & Yu, N. Selection rules for quasibound states in the continuum. *Physical Review B* **102,** 035434 (2020).
4. Ra'di, Y. & Tretyakov, S. A. Balanced and optimal bianisotropic particles: maximizing power extracted from electromagnetic fields. *New Journal of Physics* **15,** 053008 (2013).
5. Park, Y. *et al.* Violating Kirchhoff's Law of Thermal Radiation in Semitransparent Structures. *ACS Photonics* **8,** 2417–2424 (2021).
6. Máñez-Espina, L. M., Faniayeu, I., Asadchy, V. & Díaz-Rubio, A. Extreme Nonreciprocity in Metasurfaces Based on Bound States in the Continuum. *Advanced Optical Materials* **12** (1 2024).
7. Piper, J. R., Liu, V. & Fan, S. Total absorption by degenerate critical coupling. *Applied Physics Letters* **104,** 251110. ISSN: 0003-6951 (June 2014).
8. Albooyeh, M *et al.* Classification of bianisotropic metasurfaces from reflectance and transmittance measurements. *ACS Photonics* **10,** 71–83 (2023).
9. Asadchy, V. S., Díaz-Rubio, A. & Tretyakov, S. A. Bianisotropic metasurfaces: Physics and applications. *Nanophotonics* **7,** 1069–1094 (6 2018).
